# Monitoring redox stress in human airway epithelial cells exposed to woodsmoke at an air–liquid interface

**DOI:** 10.1186/s12989-024-00575-9

**Published:** 2024-03-08

**Authors:** Aiman Abzhanova, Jon Berntsen, Edward R. Pennington, Lisa Dailey, Syed Masood, Ingrid George, Nina Warren, Joseph Martin, Michael D. Hays, Andrew J. Ghio, Jason P. Weinstein, Yong Ho Kim, Earl Puckett, James M. Samet

**Affiliations:** 1 Curriculum in Toxicology and Environmental Medicine, The University of North Carolina at Chapel Hill, Chapel Hill, NC USA; 2 TRC Environmental Corporation, Raleigh, NC USA; 3 Oak Ridge Institute for Science and Education, Oak Ridge, TN USA; 4 Public Health and Integrated Toxicology Division, EPA Human Studies Facility, Research Triangle Park, 104 Mason Farm Road, Chapel Hill, NC 27599-7310 USA

## Abstract

Wildland fires contribute significantly to the ambient air pollution burden worldwide, causing a range of adverse health effects in exposed populations. The toxicity of woodsmoke, a complex mixture of gases, volatile organic compounds, and particulate matter, is commonly studied in vitro using isolated exposures of conventionally cultured lung cells to either resuspended particulate matter or organic solvent extracts of smoke, leading to incomplete toxicity evaluations. This study aimed to improve our understanding of the effects of woodsmoke inhalation by building an advanced in vitro exposure system that emulates human exposure of the airway epithelium. We report the development and characterization of an innovative system that permits live-cell monitoring of the intracellular redox status of differentiated primary human bronchial epithelial cells cultured at an air–liquid interface (pHBEC-ALI) as they are exposed to unfractionated woodsmoke generated in a tube furnace in real time. pHBEC-ALI exposed to freshly generated woodsmoke showed oxidative changes that were dose-dependent and reversible, and not attributable to carbon monoxide exposure. These findings show the utility of this novel system for studying the molecular initiating events underlying woodsmoke-induced toxicity in a physiologically relevant in vitro model, and its potential to provide biological plausibility for risk assessment and public health measures.

**Supplementary Information:**

The online version contains supplementary material available at 10.1186/s12989-024-00575-9.

## Introduction

The frequency, severity, and duration of wildland fires are increasing around the world [1–3]. Beyond the threat that they pose to life and property, wildland fires are also a major source of air pollution that impacts air quality over areas distant from their source [4, 5]. It is estimated that wildland fires contribute 30% of the ambient particulate matter (PM) burden that is responsible for excess human morbidity and mortality [6–8]. Exposure to smoke derived from wildland fires is associated with respiratory [9], cardiovascular [10, 11], immunologic[12, 13], and developmental [14, 15] health effects.

Wildland fire woodsmoke is a complex and varying mixture of gases, volatile organic compounds (VOC), and particulate matter (PM) [16, 17]. Toxicological studies of the effects of woodsmoke commonly involve exposures to resuspended PM deposited on filters or collected using a cyclone or inertial impactor [18, 19], or to extracts of smoke captured in an organic solvent [20]. Because they cannot replicate the complexity of whole smoke, these approaches provide an incomplete assessment of the combined effects of exposure to woodsmoke chemical constituents.

Being directly exposed to inhaled pollutants, the airway epithelium represents a major tissue of interest in toxicological studies of the mechanisms that underlie the health effects of woodsmoke exposure [21, 22]. In vitro studies of the toxicity of woodsmoke frequently use transformed airway epithelial cell lines cultured conventionally as a submerged cell monolayer that bears little resemblance to the airway epithelium. In contrast, when cultured at an air–liquid interface (ALI) on semipermeable membrane support, the morphology and function of primary human airway epithelial cells (pHBEC-ALI) closely approximate those of the human airway epithelium in vivo [23]. In addition, ALI conditions permit a physiologically relevant exposure of the airway epithelium to gases and aerosols.

Reversible oxidative reactions are integral to the intracellular signaling that regulates physiologic cellular processes such as energy metabolism, cell death, and gene expression [24]. Oxidative stress is commonly cited as an early event in the mechanism of action of the toxicity of environmental agents, including particulate matter and organic compounds found in woodsmoke [25–28]. The high temporal resolution of live cell microscopy is well suited to capturing transient intracellular changes in redox homeostasis events such as those caused by oxidative stress [29]. Using the genetically encoded, redox-sensitive fluorophore Grx1-roGFP2, we have previously reported dynamic changes in intracellular redox status in human airway epithelial cells exposed to oxidative air pollutants including ozone [30], zinc [31], and organic constituents found associated with ambient air PM [32, 33].

The objective of the present study was to design, fabricate, and validate an integrated imaging and exposure system that can be used to study the early oxidative effects of woodsmoke in vitro under conditions that approximate those experienced by the human airway in vivo. We report the development and characterization of a novel exposure system that permits live cell imaging of pHBEC-ALI as they are exposed directly to unfractionated woodsmoke generated in real-time. Using this system, we describe direct evidence that real-time exposure to whole woodsmoke induces dynamic oxidative changes in pHBEC-ALI.

## Materials and methods

Basic laboratory supplies and reagents were purchased from Thermo Fisher Scientific (Waltham, MA). Minimum essential media with GlutaMAX™ (MEM GlutaMAX™), Hanks’ balanced salt solution (HBSS) with calcium and magnesium, penicillin–streptomycin solution, and fetal bovine serum (FBS) were purchased from Gibco (Waltham, MA), and bovine type 1 collagen was obtained from Thermo Fisher Scientific. Hydrogen peroxide (H_2_O_2_), dithiothreitol (DTT), 2-acetylamino-3-[4-(2-acetylamino-2-carboxyethylsulfanylthiocarbonylamino) phenylthiocarbamoylsulfanyl] propionic acid (2-AAPA) were purchased from Sigma-Aldrich (St Louis, MO). Locke’s solution was prepared in-house and buffered to pH 7.4 as described previously [30]. All mass flow controllers were purchased from Teledyne-Hastings (Hampton, VA).

### Culture of primary cells expressing Grx1-roGFP2

Primary human bronchial epithelial cells (pHBEC) were obtained from healthy volunteers during bronchoscopy, following a protocol and consenting materials approved by the University of North Carolina School of Medicine Committee on the Protection of the Rights of Human Subjects and by the US Environmental Protection Agency. The cells were expanded and then grown on 12 mm Transwell™ inserts with a pore size of 0.4 um (Costar; Tewksbury, MA) according to previously described protocols [34]. The genetically encoded fluorescent redox sensor, Grx1-roGFP2, was expressed in pHBEC as described previously [30, 32, 33, 35]. pHBEC were seeded at a density of 200,000 cells per Transwell™ insert and were cultured submerged until confluent. Following the removal of apical media, the cells were maintained at ALI for a minimum of 21 days before use in experiments [36]. Cells were cultured at 37 °C in a humidified environment containing 5% carbon dioxide (CO_2_). Media was changed 3 times per week.

### Live cell exposure to freshly generated wood smoke and imaging

The tube furnace was adapted from a design as described previously [37]. The system shown is composed of three units: A. Smoke generation; B. Smoke conditioning; and.

**Fig. 1:**
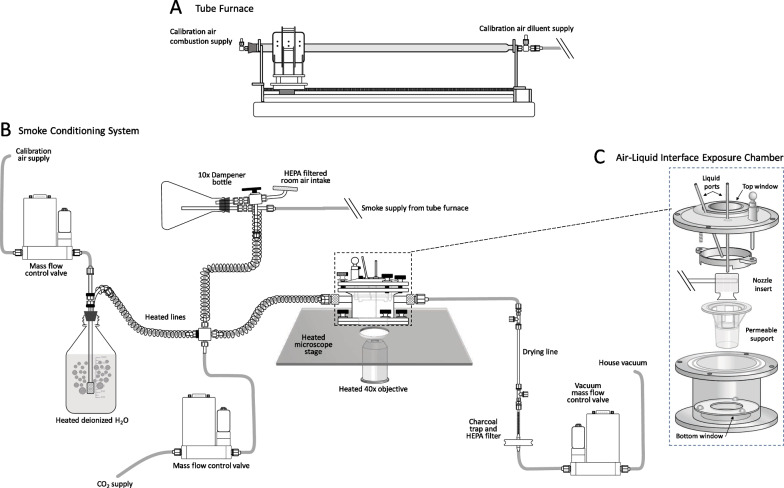
Live-cell imaging system for exposure of pHBEC-ALI exposed to woodsmoke produced in real-time. (**A**) Red oak fragments (500–2000 um) are heated to 550 °C using a ceramic ring heater moving over a quartz tube. Calibration-grade air is supplied through the furnace at a rate of 4 L/min. The resulting woodsmoke is diluted with and carried by an additional 50 L/min flow of calibration-grade air. (**B**) A flow of 20 mL/min diluted smoke is brought under negative pressure into a 300 mL bottle and mixed with 170 mL/min of humidified air and 10 mL/min (5%) CO_2_ before entering a stage-top imaging and exposure chamber holding pHBEC-ALI cultured on a 12-mm Transwell™ insert (**C**). In the exposure chamber, the intensity of 510 nm fluorescence induced by sequential excitation of pHBEC-ALI expressing Grx1-roGFP2 with 408 nm and 488 nm is monitored at 40X magnification with 60-s resolution. All Teflon and stainless-steel components are heated to prevent condensation

C. Exposure and imaging (see Fig. 1).

Red oak (*Quercus rubra*) dowels with a 6.35 mm (¼ inch) diameter (Amazon, Seattle, WA) were broken into 10–20 cm pieces, shredded in a blender (Blendtec, Orem, UT), and then sieved through a stack of meshes with U.S. Standard No. 5 (4000 microns), No. 10 (2000 micron), and No. 35 (500 microns) (Wildco, Yulee, FL). Fragments sized between 500 and 2000 um (shredded red oak) were stored at room temperature and used as fuel for the furnace. For a standard run, the tube furnace (Fig. 1A) was loaded with 22.5 g of shredded red oak fuel, spread over 20.32 cm (8 inches) in linear dimension. The heater was set to 550 °C and advanced at a rate of 0.05 mm/sec, yielding an average fuel consumption of 0.33 g/min. Calibration-grade air (CGA) was derived by compressing and purifying ambient air to medical air standards, followed by removal of ozone (O_3_), nitrogen oxides (NO_x_), carbon monoxide (CO), sulfur dioxide (SO_2_), and hydrocarbons with an air purification system (AADCO, Village of Cleves, OH). Combustion was supported by a controlled flow of CGA at 4 L/min (Teledyne Model: HFC-202). The resulting smoke was diluted 1:13 by a flow of 50 L/min of room temperature CGA (Teledyne Model: HFC-203). In some experiments the woodsmoke was diluted with a 25 L/min flow (1:7 dilution). The diluted smoke passed through 2.54 cm diameter (OD) stainless-steel tubing in a circuit that returned to the fume hood past the conditioning system.

A 300 mL bottle was placed at the entrance to the conditioning system to dampen the oscillations in the wood smoke mass concentration. Room-temperature diluted smoke was brought into the conditioning system under negative pressure using a vacuum mass-flow controller (Teledyne Model: HFC-D-302B) set to 200 mL/min. A flow of 5% (10 mL/min) CO_2_ was supplied by a mass flow controller (Teledyne Model: HFC-D-302B). CGA flow (Teledyne Model: HFC-202) at 170 mL/min was diffused through 38 °C deionized sterile water. The temperature of the water and heated lines was adjusted to produce ≥ 90% relative humidity at 37 °C at the entrance to the chamber. All stainless steel and Teflon lines were heated to prevent condensation (BriskHeat, Columbus, OH, Fig. 1B).

pHBEC-ALI expressing the Grx1-roGFP2 sensor were equilibrated for 2 h in glucose-free Locke’s buffer. Thirty minutes prior to the experiment, cells were transferred to a custom-built stainless-steel imaging chamber (Fig. 1C) containing 3 mL of glucose-free Locke’s buffer in the basolateral compartment. The chamber was equipped with inlet and outlet Luer lock couplings and a lid fitted with a stainless steel ring to support a 12 mm Transwell™ insert (Corning, Corning, NY). The smoke was introduced into the chamber through a 1/8-inch stainless steel blunt needle with a slit aimed at the cell culture surface. A trumpet-shaped Lexan (polycarbonate resin thermoplastic) insert (Fig. 1C inset) directed the flow of the mix of particles, vapor, and gases 2 mm above the cell culture surface. Openings at the top and bottom of the chamber accommodate #1.5 optical grade glass coverslips for widefield and confocal microscopy.

### Microscopy

Time-series experiments of pHBEC -ALI expressing Grx1-roGFP2 were conducted at a temporal resolution of 1 acquisition/minute. All live-cell imaging experiments were performed using a Nikon Eclipse C1si spectral confocal imaging system using 405 nm, and 488 nm primary lasers with a 40 × ELWD objective (Nikon Instruments, Melville, NY). Fluorescence emission was detected using a 523/30 nm band-pass filter (Chroma, Bellows Falls, VT). Laser power and pixel dwell time remained consistent throughout all experiments, while detector gain was optimized before the start of each experiment. Regions of interest (ROI) were drawn for 10 individual cells and monitored for the duration of the experiment. Fluorescence emission ratios (405/488) were calculated and normalized to the baseline and maximal response for each cell. The normalized ratios for individual ROIs were averaged and plotted as mean ± standard error of the mean (± SEM).

### Smoke characterization

Carbon dioxide (CO_2_) levels were measured using CO_2_ gas analyzer Viasensor G100-10N (Viasensor, Cocoa, FL). Carbon monoxide (CO) levels were measured using CO analyzer Model: 48iQ CO; (Thermo Scientific, Franklin, MA). Nitrogen oxides (NO, NO_2_, NO_x_) levels were measured with chemiluminescent analyzer model 42i/42iQ NO, NO_2_, NO_x_ (Thermo Scientific). Wood smoke PM was collected on pre-weighed Teflon™ filters and the weight difference was normalized to the volume of air. For the Scanning Electron Microscopy (SEM), samples were prepared in an ULPA filtered positive-pressure laminar flow clean bench. The 47 mm Teflon™ filter samples were adhered to 25.4 mm SEM pin stubs with double coated carbon conductive tabs. The SEM sample stubs were carbon coated with a Cressington 208C coater to 20 nm thickness. Samples were analyzed with a Tescan MIRA3 field emission scanning electron microscope operated at 7 keV and images were captured using the secondary electron detector at a working distance of approximately 5 mm. Particle size distribution in the range of 17 nm to 1000 nm was measured using a scanning mobility particle sizer (SMPS, Model 3938, TSI Inc., Shoreview, MN). The SMPS instrument also provided the number concentration of submicron particles. The ratio of organic carbon to elemental carbon (OC/EC) was analyzed by collecting smoke samples on a prebaked quartz filter and analyzing 1.5 cm^2^ punches using a thermal-optical analyzer (107A; Sunset Laboratory Inc, Hillsborough, NC). For inorganic elemental analysis, samples collected on Teflon filters were digested using 1 mL trace metal grade concentrated nitric acid (HNO_3_) (Thermo Fisher Scientific), heated to 70 °C for 24 h, and centrifuged. The resulting supernatant was analyzed using Inductively Coupled Plasma-Optical Emission Spectroscopy (ICP-OES) (Optima 4300D, PerkinElmer, Norwalk, CT). Air samples were collected from the tube furnace using ceramic-coated stainless steel evacuated canisters to measure a range of speciated VOCs and using 2,4-dinitrophenylhydrazine (DNPH)-coated silica cartridges (PN 505323, Sigma-Aldrich Co., St. Louis, MO) to quantify carbonyl compounds. After sampling was completed, the canister samples were analyzed by gas chromatograph coupled to a mass spectrometer (GC–MS) in accordance with EPA method TO-15 as previously described [38]. The DNPH cartridges were extracted with 6 mL of carbonyl-free acetonitrile after sampling and analyzed by high-performance liquid chromatography following EPA method TO-11A.

For sVOC sampling and analysis, a 0.385 cm^2^ punch from an ashed quartz fiber filter was placed in a conditioned glass tube (178 mm total length, 6 mm O.D., 4 mm I.D. with a glass frit 15 mm from the upstream end and packed with 40 mm of Carbotrap F and 20 mm of Carbotrap C) such that sample air passed through the filter. The sample air, not treated after relevant experimental conditions, was collected at 50 mL/min. The sample tube was stored in a sealed container at 4 °C until analysis. During analysis, the tube was desorbed at 300 °C for 11 min while being purged with 50 mL/min He(g) in the direction opposite of flow during sample collection using a Gerstel TDS3 thermal desorption device with a TDSA2 autosampler (Gerstel, Linthicum, MD). The desorbed analytes were collected on a CIS4 inlet/cold trap installed on an Agilent 6890a GC system (Agilent, Santa Clara, CA) with HP-5 ms-UI column (30 m, 250 um ID, 0.25 um film, Agilent). This trap was maintained at −100 °C throughout the desorption. When desorption was complete, the CIS4 was heated to 300 at 12 °C per minute during the GC run (10 °C min^−1^ from 65 to 300 °C where it is held for 15 min). Analyte detection took place on an Agilent 5973 mass selective detector operating in EI-Scan mode, scanning the mass range from 44 to 500 m/z.

### Statistical analysis

All imaging data were quantified using NIS-Elements AR software (Nikon). Statistical analysis and graphs were conducted using PRISM (GraphPad Software, La Jolla, CA). Pairwise comparison was carried out using Student’s t-test. Statistical significance was assigned at *p* < 0.05.

## Results

To simulate in vivo woodsmoke exposure conditions experienced by human airway epithelial cells in an in vitro model, we first constructed a system that conditions unfractionated woodsmoke and renders it biocompatible with pHBEC-ALI. Woodsmoke generated by combusting red oak in a tube furnace was diluted with heated and humidified air and mixed with a flow of 5% CO_2_ before flowing past pHBEC-ALI in a heated stage-top imaging chamber.

The gaseous and particulate components in woodsmoke generated by the combustion of red oak fragments in a tube furnace were characterized using a variety of quantitative analytical methods. Table 1 summarizes the inorganic and organic composition of woodsmoke produced by the combustion of 500–2000 um red oak fragments at 550 °C, as supported by a 4 L/min flow of CGA through the furnace and diluted by a standard flow of 50 L/min of CGA introduced as a carrier. SEM of captured wood smoke particles show discrete spheres with a size range that approximates the geometric mean particle diameter reported by SMPS analyses (Table 1, Additional file 1: Fig. S1). Additional file 7: Table S7 and Additional file 8: Table S8 show concentrations of volatile (VOC) and semi-volatile organic compounds (sVOC), respectively, identified in samples of whole smoke under the same standardized conditions. Consistent with previous reports of the composition of oak combustion emissions [39], major VOC components included methanol, acetaldehyde, formaldehyde, 3-furaldehyde, 2-methyl furan, acrolein, acetone, and propylene. Also as expected [40, 41], the sVOC analyses were dominated by the presence of methoxyphenol compounds. Additional file 9: Table S9 summarizes the levels of metals found in wood smoke samples.

**Table 1:** Characteristics of red oak woodsmoke generated in the tube furnace

Characteristics (unit)	At the Furnace	At the chamber (1:10)
PM (mg/m^3^) ± SD	513 ± 22	51.3
CO (ppm)	1400	140
CO_2_ (ppm)	1600	160
NO (ppm)	0.56	0.056
NO_2_ (ppm)	0.04	0.004
NOx (ppm)	0.6	0.06
OC/EC	1048	–
Geo. Mean particle diameter (nm) ± Std*	–	336.3 ± 1.5
Particle count (particles/cm^3^)*	–	2.78 × 10^7^

*As reported by SMPS

To assess the system’s ability to maintain cell viability and the stability of intracellular redox homeostasis over the course of the experimental period, pHBEC-ALI expressing Grx1-roGFP2 were exposed to conditioned emissions flowing through the system while operating with no fuel (control) in the tube furnace. As shown in Additional file 2: Fig. S2, no significant changes in redox homeostasis from baseline were observed, as reported by the normalized Grx1-roGFP2 fluorescence, throughout the 50-min control exposure. The expected maximal responses to the additions of the 10 mM H_2_O_2_ and 20 mM DTT, the oxidative and reductive stimuli, respectively, confirmed that the cells remained fully responsive at the end of the experiment (Additional file 2: Fig. S2). Taken together, these findings demonstrated that the airflow, pH, humidity, and temperature provided by the system are compatible with normal pHBEC-ALI stability and redox homeostasis for the duration of the experimental exposures.

**Fig. 2:**
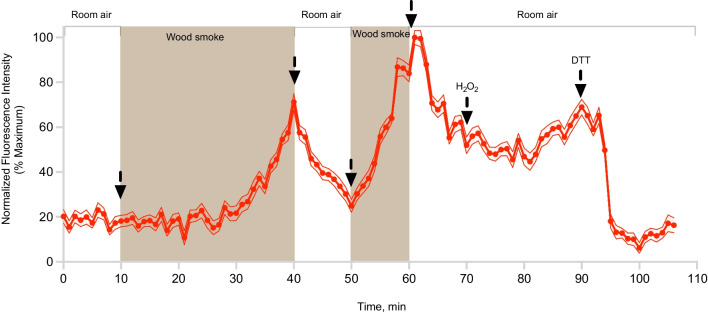
Oxidative stress in pHBEC-ALI exposed to woodsmoke. Prior to the study, pHBEC-ALI expressing Grx1-roGFP2 were deprived of glucose for 2 h. Woodsmoke or filtered room air was conditioned and introduced into the chamber at the indicated times. The intensity of 510 nm fluorescence induced by sequential excitation of the cells with 408 nm and 488 nm is monitored at 40X magnification with 60-s resolution. Control additions of 10 mM H_2_O_2_ and 20 mM DTT were made at the end of the exposure period. Plotted is the normalized fluorescence intensity expressed as the ratio of emissions from 405 and 488 nm excitation. These results represent five separate experiments, with values presented as a mean ± SEM for 10 individual cells

As shown in Fig. 2, the introduction of woodsmoke into the air stream flowing through chamber resulted in a time-dependent, sharp increase in Grx1-roGFP2 oxidation in pHBEC-ALI over a 15-min period (See Additional file 3: Fig. S3 for representative images). Notably, this oxidative response was rapidly reversed when woodsmoke exposure was interrupted and replaced by a flow of filtered room air, demonstrating functionally that pHBEC-ALI remained viable at that point in the exposure. The reintroduction of smoke into the air stream caused an earlier and larger increase in Grx1-roGFP2 oxidation which was reversible again by changing to filtered room air. Following these repeated cycles of woodsmoke and clean air exposures, stimulation with H_2_O_2_ had a muted effect, while the response to DTT aligned with the expected return to baseline (Fig. 2).

The dose-dependency of the oxidative response to woodsmoke was examined in pHBEC-ALI expressing Grx1-roGFP2 exposed to two different dilutions of woodsmoke generated in the tube furnace using the same mass of red oak and combustion conditions. Compared to pHBEC-ALI exposed to a lower woodsmoke concentration, those exposed to a twofold higher concentration showed a more rapid and accelerated oxidative response, resulting in a higher normalized signal intensity (Table 2, Additional file 4: Fig. S4).

**Table 2:** Dose-dependency of the oxidative response to red oak woodsmoke exposure in pHBEC-ALI

	Higher dose (4 + 25 L/min)	Standard dose (4 + 50 L/min)
Time-to-response, min ± Std	15 ± 5.7	28.2 ± 6.4
Max n-fold change ± Std	3 ± 1.5	1.55 ± 0.3
PM mass, mg/m^3^ ± Std	835 ± 17	513 ± 22

**Fig. 3:**
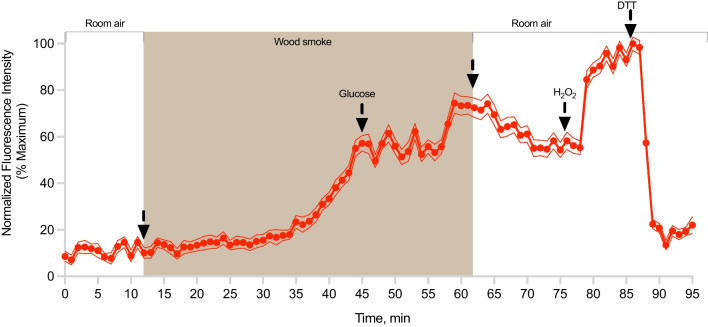
Glucose does not reverse oxidation of the Grx1-roGFP2 sensor in pHBEC-ALI following exposure to woodsmoke. Changes in the ratio of oxidized and reduced glutathione induced by exposure to freshly generated wood smoke in primary bronchial epithelial cells cultured at air–liquid interface (pHBEC-ALI) followed by the addition of glucose

We have previously shown that availability of glucose can rapidly restore NADPH levels and reverse roGFP2-reported oxidative changes in airway epithelial cells exposed to an environmental peroxide [32, 33]. Therefore, we next determined the effect of glucose on woodsmoke-induced oxidative stress in glucose-deprived pHBEC-ALI. As shown in Fig. 3 and Additional file 5: Fig. S5, the addition of 1 mM glucose appeared to arrest, but not reverse, the development of Grx1-roGFP2 oxidation induced by exposure of pHBEC-ALI to woodsmoke.

Glucose does not reverse the oxidation of Grx1-roGFP2 in cells exposed to wood smoke. Prior to exposure, pHBEC-ALI expressing Grx1-roGFP2 were deprived of glucose for 2 h. The exposure started after a 10-min baseline at the indicated time and continued for 50 min (shaded area), after which cells were exposed to filtered room air. At 45 min mark, 1 mM glucose was added basolaterally. Controls additions of 10 mM H_2_O_2_ and 20 mM DTT were made at indicated times. Emitted fluorescence intensity values were normalized to the baseline and maximum response. The results shown are representative of five separate experiments. Values represent a mean ± SEM for 10 individual cells.

Carbon monoxide can inhibit mitochondrial electron transport [42], which could potentially result in an accumulation of partially reduced oxygen species, leading to oxidative stress in pHBEC-ALI. To investigate whether the oxidative effect of woodsmoke exposure could be attributed to CO exposure, we next exposed pHBEC-ALI expressing Grx1-roGFP2 to the concentration of CO equivalent to that measured in the woodsmoke. pHBEC-ALI exposed to a final concentration of 230 ppm CO for 40 min showed no change in redox status, as reported by the Grx1-roGFP2 sensor. As anticipated, the effects of H_2_O_2_ and DTT controls, introduced at the end of the CO exposure for maximal and minimal deflections in normalized signal intensity, were consistent with expectations (Additional file 6: Fig. S6).

## Discussion

In vitro studies provide valuable mechanistic understanding of the effects of human exposure to environmental pollutants such as woodsmoke. However, the interpretation of findings from studies using conventional in vitro approaches is constrained by multiple experimental factors, including the use of transformed epithelial cell lines, which lack the complexity, morphology, and function of the pseudostratified columnar ciliated epithelium covering the airway [43, 44]. These cell lines are typically cultured and exposed as a monolayer covered by a column of aqueous medium that may be several millimeters deep, raising questions about the delivered dose of particles and gases, as well as the solubility of woodsmoke constituents in aqueous medium and their potential quenching by media components. In developing the system described in this report, we sought to avoid these limitations by designing it to support primary human airway cells cultured under physiologic ALI conditions. ALI culture also confers the added advantage of recapitulating in vivo exposure to inhaled woodsmoke, where particles deposit and gases diffuse through a physiological layer of airway surface liquid and mucus produced by the epithelial cells that is only 50 μm in depth [44–46]. By incorporating ALI conditions, our system provides a more physiologically relevant model to investigate the cell responses to wood smoke.

While useful in some experimental applications*, *in vitro exposure to smoke particles or smoke condensate dissolved in an organic medium cannot capture the complex composition of real woodsmoke produced by wildland fires. By interfacing the exposure and imaging chamber with a tube furnace, the exposure system is alimented by a controllable and reproducible flow of woodsmoke with a relatively constant and reproducible composition that remains stable during the experimental exposure period. This allows for time-dependent cumulative dosing of the cells that simulates exposure of the airway epithelium during inhalational exposure to woodsmoke. Capturing unfractionated furnace emissions in real time ensures that the cells are exposed to a more complete representation of woodsmoke gases, particulates, and volatile and semi-volatile organic compounds.

In this study, we present direct evidence of intracellular oxidative stress caused by exposure to woodsmoke in pHBEC-ALI. Oxidative stress is arguably the most commonly cited feature of the toxicity of environmental agents, including that of metals [26], woodsmoke gases such as NO_2_, electrophilic VOC like acrolein, and redox-active semi-volatile quinones [47–49]. The Grx1- roGFP2 sensor used in this study equilibrates with the intracellular glutathione pool through a redox relay initiated by peroxides and enzymatically mediated by glutathione peroxidases and glutaredoxin [24, 50]. It is also possible, however, that the spectral change in Grx1- roGFP2 that we reported in pHBEC-ALI exposed to woodsmoke is caused by a direct attack on cysteinyl residues of the sensor by electrophiles present in woodsmoke. The volatile organic analyses performed in this study show the presence of a number of reactive aldehydic compounds (e.g., acrolein) capable of electrophilic adduction of thiol targets. In addition, secondary formation of organic peroxides may plausibly lead to roGFP2 oxidation through the redox relay. Additional studies are needed to determine whether the oxidative stress reported in pHBEC-ALI exposed to woodsmoke reflects intracellular peroxidative glutathione oxidation, electrophilic oxidation of Grx1-roGFP2, or a combination of these two potential mechanisms.

The intracellular oxidative stress reported by Grx1-roGFP2 in pHBEC-ALI exposed to woodsmoke showed a remarkable rate of recovery upon cessation of the exposure, which was shown to be repeatable for at least two cycles during a 60-min experiment (Fig. 2). This recovery is not only functional evidence of the viability of the pHBEC-ALI during exposure to woodsmoke, it also offers insights into the adaptive mechanisms at work within the cells as they undergo exposure to woodsmoke. Glutathione oxidation reported by Grx1-roGFP2 exposed to peroxidative stimuli is reversed by glutathione reductase at the expense of NADPH [35, 51, 52]. On the other hand, it is possible that electrophilic oxidation of cysteinyl thiols forms a disulfide in Grx1-roGFP2 that is subsequently reduced by other antioxidant enzymes such as protein disulfide isomerase [53], glutathione-S-transferase [54], or glutaredoxin [55]. In this regard, the observation that the addition of glucose can arrest the oxidation of Grx1-roGFP2 in pHBEC-ALI exposed to woodsmoke, but not reverse it in the face of continued exposure suggests that woodsmoke-induced intracellular oxidative stress involves an interplay of multiple mechanisms (Fig. 3, Additional file 5: Fig. S5).

This is the first study reporting the development and characterization of a live-cell imaging system for the exposure of pHBEC-ALI to unfractionated woodsmoke. Limitations include the use of only one type of fuel (dry red oak) using a single smoldering, non-flaming, temperature. In addition, the rate of movement of the heater over the fuel and the flow of supply air during the combustion were not varied. It is, therefore, likely that the generalizability of the combustion emissions characterized in this study is limited. Ongoing work will enhance our understanding of the performance of this system and show its potential as a tool for the investigation of the cellular, biochemical, and molecular mechanisms that underlie the health effects of woodsmoke inhalation.

## Conclusions

In summary, we report the development and characterization of an innovative imaging and exposure system that avoids multiple limitations that afflict current in vitro approaches to the study of the toxicology of woodsmoke. We demonstrate its utility in monitoring intracellular redox perturbations in fully differentiated human airway epithelial cells undergoing exposure to whole woodsmoke emissions generated in real time. The system offers promise as a risk assessment tool to elucidate the molecular initiating and key events in the health effects of woodsmoke exposure and identify strategies for their potential mitigation.

### Supplementary Information

**Additional file 1**. **Fig. S1**. Scanning electron micrograph of red oak wood smoke particles Scanning electron microscope (SEM) image of particles produced by the tube furnace under conditions described in Methods captured on a Teflon^TM^ filter (12800 magnification).

**Additional file 2**. **Fig. S2**. Exposure to system emissions alone does not initiate oxidative responses in pHBEC-ALI. Control exposure to the heated empty tube furnace and system air does not initiate the oxidation of Grx1-roGFP2. Prior to exposure, primary bronchial epithelial cells cultured at air-liquid interface (pHBEC-ALI) expressing Grx1-roGFP2 were deprived of glucose for 2 hours. The cells were then exposed to system air following a 10-minute baseline at the indicated time and were exposed to 50 minutes (shaded area) before control additions of 10 mM H_2_O_2_ and 20 mM DTT were done at indicated times to verify the sensitivity of the assay. Emitted fluorescence intensity values were normalized to the baseline and maximum response. The results presented are representative of three separate experiments, with values representing a mean ± SEM for 10 individual cells.

**Additional file 3**. **Fig. S3**. Changes in fluorescence intensity of the Grx1-roGFP2 redox sensor showing the response of pHBEC-ALI to oxidative and reductive stress. pHBEC-ALI were exposed to wood smoke, 10 mM H_2_O_2_, and 20 mM DTT. Images were collected from a representative wood smoke exposure experiment.

**Additional file 4**. **Fig. S4**. Wood smoke induces oxidation of the Grx1-roGFP2 redox sensor in pHBEC-ALI dose-dependently pHBEC-ALI expressing the Grx1-roGFP2 were deprived of glucose for 2 hours before exposure. Wood smoke exposure started at 10-minute mark following baseline measurement. The air supply was changed to filtered room air at 25-minute mark for the higher dose (blue circles), and at 30-minute mark for the standard dose (red triangles). Basolateral addition of H_2_O_2_ was done at 40-minute mark. DTT was added at indicated times. Values for each line represent a mean ± SEM for 10 individual cells.

**Additional file 5**. **Fig. S5**. Comparison of responses to wood smoke in glucose deprived and glucose treated pHBEC-ALI expressing Grx1-roGFP2 pHBEC-ALI expressing the Grx1-roGFP2 were deprived of glucose for 2 hours before the experiment. Exposure to wood smoke followed a 10-minute baseline. Wood smoke was introduced at the indicated time. At 50 minute, 1 mM glucose (blue circle) or vehicle (red triangle) was added basolaterally and the smoke was switched to filtered room air. Values for each line represent a mean ± SEM for 10 individual cells.

**Additional file 6**. **Fig. S6**. Carbon monoxide at a concentration found in woodsmoke does not initiate the oxidation of Grx1-roGFP2 Carbon monoxide (CO) at a concentration found in freshly generated smoke does not initiate the oxidation of Grx1-roGFP2. Prior to exposure, primary bronchial epithelial cells cultured at air-liquid interface (pHBEC-ALI) expressing Grx1-roGFP2 were deprived of glucose for 2 hours. The cells were then exposed to 230 ppm CO after a 10-minute baseline at the indicated time and were exposed to 30 minutes (shaded area) before being exposed to filtered room air. Control additions of 10 mM H_2_O_2_ and 20 mM DTT were done at indicated times to verify the sensitivity of the assay. Emitted fluorescence intensity values were normalized to the baseline and maximum response. The results presented are representative of three separate experiments, with values representing a mean ± SEM for 10 individual cells.

**Additional file 7**. **Table S1**. Concentrations (ppbv) of volatile organic compounds (N = 154) in the red oak smoke samples.

**Additional file 8**. **Table S2**. Semi-volatile organic compounds (N = 88) measured in two red oak smoke samples collected on separate days. * Compound could not be unambiguously identified (N.D.); ** Values below reliable quantification (> Quant)

**Additional file 9**. **Table S3**. Concentration (ppm) of inorganic elemental constituents of red oak smoke. *Elemental concentrations of Zn, Ni, Mn, V, Cu, and Mg are not detectable

## Abbreviations

GSH: Reduced glutathione
GSSG: Oxidized glutathione
GSSG: GSH: Ratio of oxidized glutathione to reduced glutathione
pHBEC: Primary human bronchial epithelial cells
ALI: Air–liquid interface
PM: Particulate matter
VOC: Volatile organic compound
sVOC: Semi-volatile organic compound
CGA: Calibration-grade air
roGFP: Reduction–oxidation sensitive green fluorescent protein
Grx: Glutaredoxin
SMPS: Scanning mobility particle sizer
SEM: Scanning electron microscope
