# Supplementary figures and images for: Monitoring redox stress in human airway epithelial cells exposed to woodsmoke at an air–liquid interface
Source: Part Fibre Toxicol. 2024 Mar 8;21:14. doi: 10.1186/s12989-024-00575-9 (PMC10921608; doi:10.1186/s12989-024-00575-9)

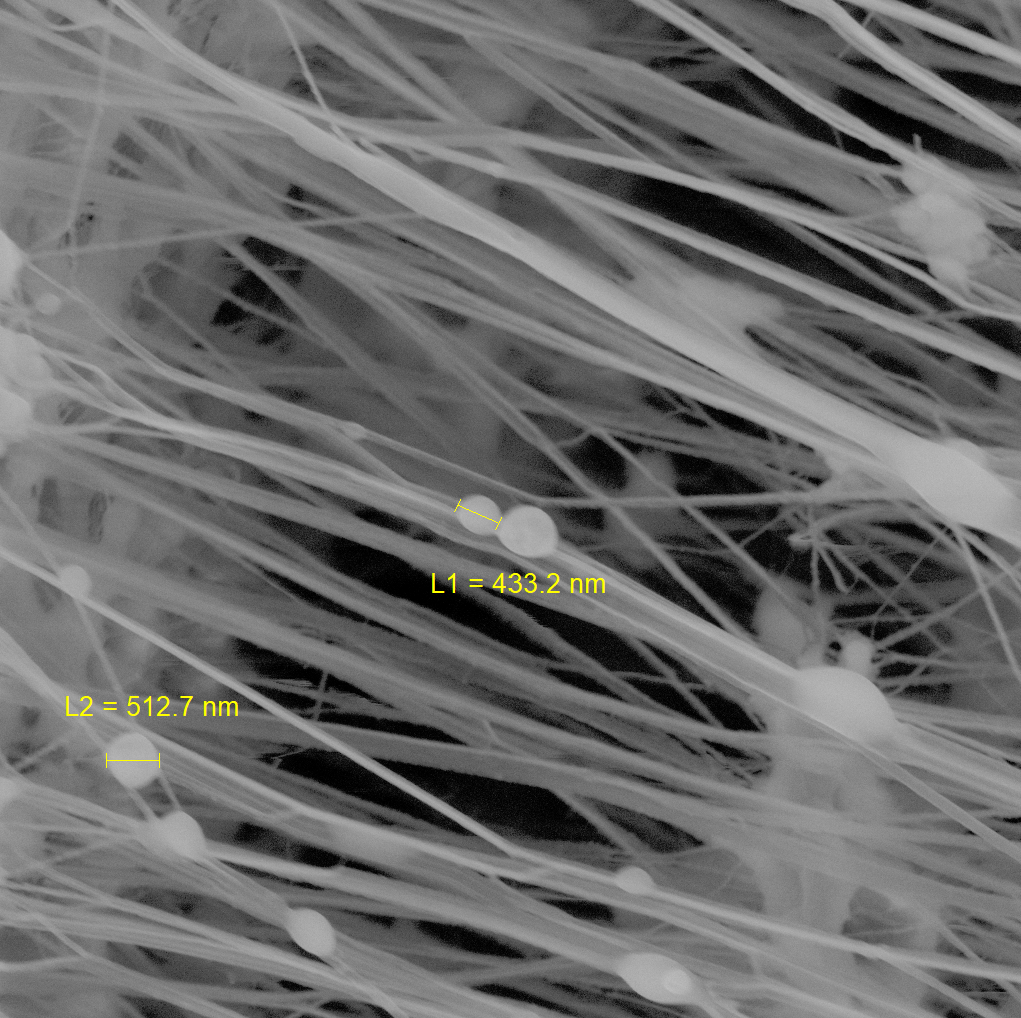

Supplement: Supplementary file 1 — Additional file 1. Fig. S1. Scanning electron micrograph of red oak wood smoke particles Scanning electron microscope (SEM) image of particles produced by the tube furnace under conditions described in Methods captured on a TeflonTM filter (12800 magnification). [file 12989_2024_575_MOESM1_ESM.tif]

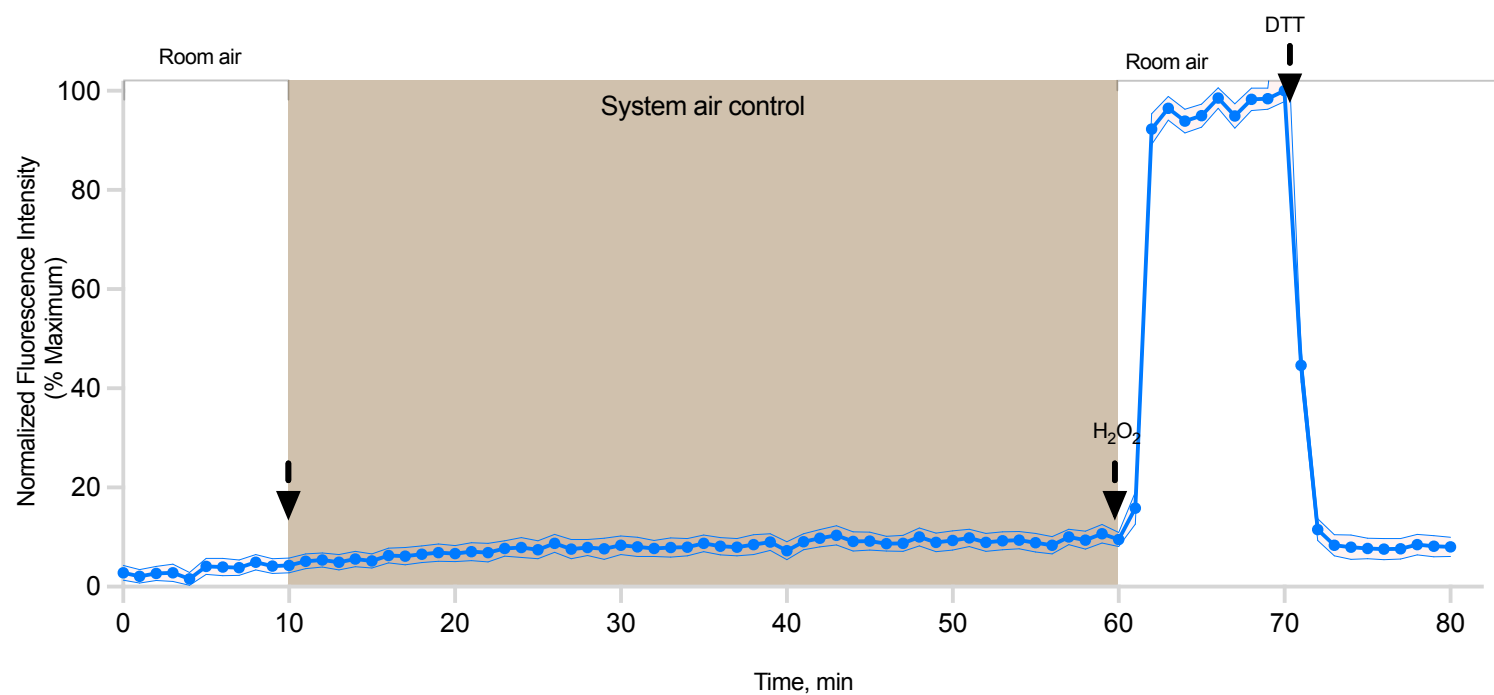

Supplement: Supplementary file 2 — Additional file 2. Fig. S2. Exposure to system emissions alone does not initiate oxidative responses in pHBEC-ALI. Control exposure to the heated empty tube furnace and system air does not initiate the oxidation of Grx1-roGFP2. Prior to exposure, primary bronchial epithelial cells cultured at air-liquid interface (pHBEC-ALI) expressing Grx1-roGFP2 were deprived of glucose for 2 hours. The cells were then exposed to system air following a 10-minute baseline at the indicated time and were exposed to 50 minutes (shaded area) before control additions of 10 mM H2O2 and 20 mM DTT were done at indicated times to verify the sensitivity of the assay. Emitted fluorescence intensity values were normalized to the baseline and maximum response. The results presented are representative of three separate experiments, with values representing a mean ± SEM for 10 individual cells. [file 12989_2024_575_MOESM2_ESM.pdf]

405/488

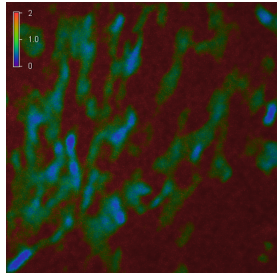

Baseline

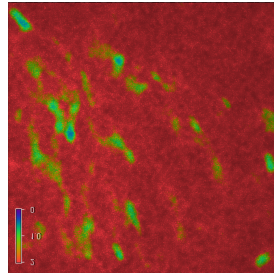

Wood smoke

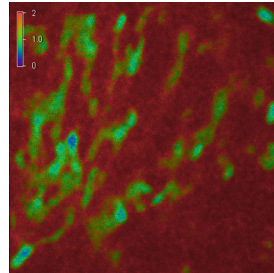

$H_2O_2$

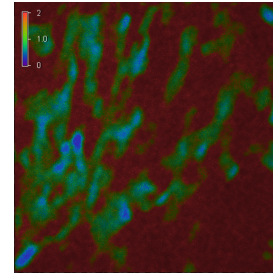

DTT

Supplement: Supplementary file 3 — Additional file 3. Fig. S3. Changes in fluorescence intensity of the Grx1-roGFP2 redox sensor showing the response of pHBEC-ALI to oxidative and reductive stress. pHBEC-ALI were exposed to wood smoke, 10 mM H2O2, and 20 mM DTT. Images were collected from a representative wood smoke exposure experiment. [file 12989_2024_575_MOESM3_ESM.pdf]

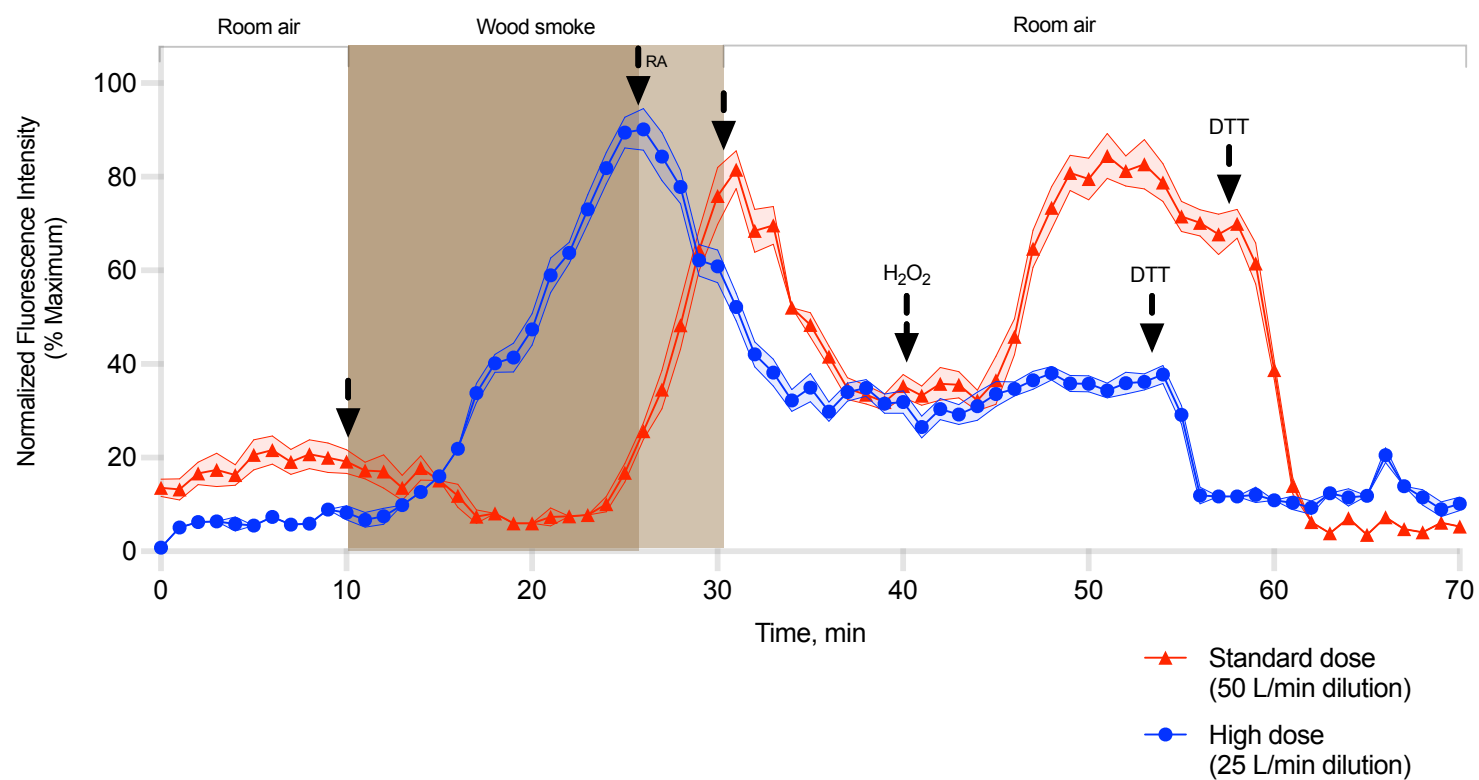

Supplement: Supplementary file 4 — Additional file 4. Fig. S4. Wood smoke induces oxidation of the Grx1-roGFP2 redox sensor in pHBEC-ALI dose-dependently pHBEC-ALI expressing the Grx1-roGFP2 were deprived of glucose for 2 hours before exposure. Wood smoke exposure started at 10-minute mark following baseline measurement. The air supply was changed to filtered room air at 25-minute mark for the higher dose (blue circles), and at 30-minute mark for the standard dose (red triangles). Basolateral addition of H2O2 was done at 40-minute mark. DTT was added at indicated times. Values for each line represent a mean ± SEM for 10 individual cells. [file 12989_2024_575_MOESM4_ESM.pdf]

Normalized Fluorescence Intensity  
(% Maximum)

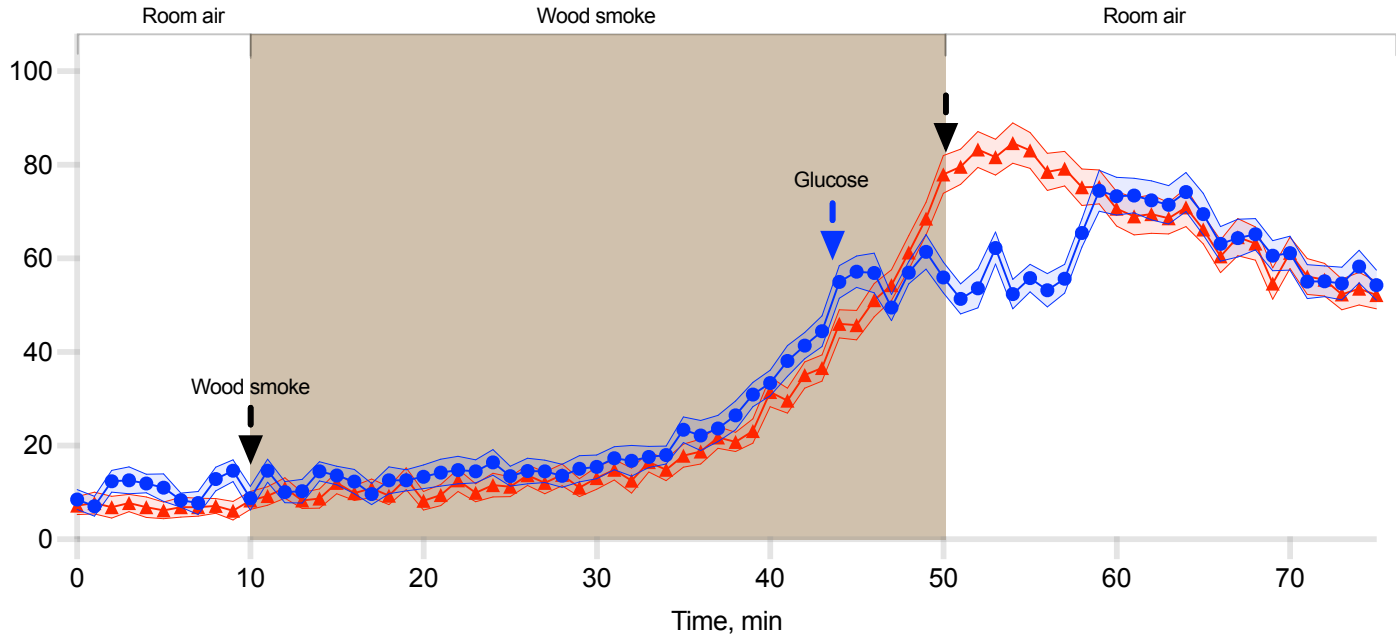

—●— with glucose added

—▲— no glucose

Supplement: Supplementary file 5 — Additional file 5. Fig. S5. Comparison of responses to wood smoke in glucose deprived and glucose treated pHBEC-ALI expressing Grx1-roGFP2 pHBEC-ALI expressing the Grx1-roGFP2 were deprived of glucose for 2 hours before the experiment. Exposure to wood smoke followed a 10-minute baseline. Wood smoke was introduced at the indicated time. At 50 minute, 1 mM glucose (blue circle) or vehicle (red triangle) was added basolaterally and the smoke was switched to filtered room air. Values for each line represent a mean ± SEM for 10 individual cells. [file 12989_2024_575_MOESM5_ESM.pdf]

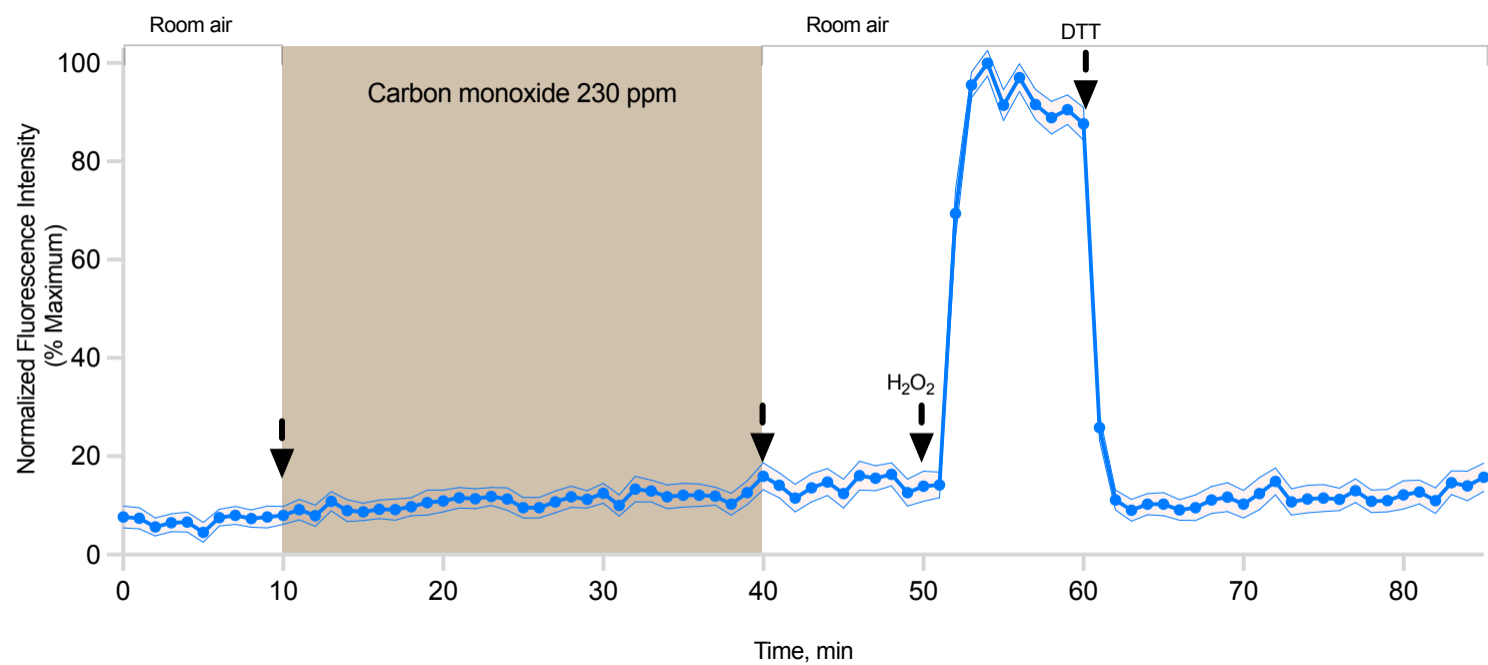

Supplement: Supplementary file 6 — Additional file 6. Fig. S6. Carbon monoxide at a concentration found in woodsmoke does not initiate the oxidation of Grx1-roGFP2 Carbon monoxide (CO) at a concentration found in freshly generated smoke does not initiate the oxidation of Grx1-roGFP2. Prior to exposure, primary bronchial epithelial cells cultured at air-liquid interface (pHBEC-ALI) expressing Grx1-roGFP2 were deprived of glucose for 2 hours. The cells were then exposed to 230 ppm CO after a 10-minute baseline at the indicated time and were exposed to 30 minutes (shaded area) before being exposed to filtered room air. Control additions of 10 mM H2O2 and 20 mM DTT were done at indicated times to verify the sensitivity of the assay. Emitted fluorescence intensity values were normalized to the baseline and maximum response. The results presented are representative of three separate experiments, with values representing a mean ± SEM for 10 individual cells. [file 12989_2024_575_MOESM6_ESM.pdf]
