# Supplementary material for: Monitoring redox stress in human airway epithelial cells exposed to woodsmoke at an air–liquid interface
Source: Part Fibre Toxicol. 2024 Mar 8;21:14. doi: 10.1186/s12989-024-00575-9 (PMC10921608; doi:10.1186/s12989-024-00575-9)
Supplement: Supplementary file 7 — Additional file 7. Table S1. Concentrations (ppbv) of volatile organic compounds (N = 154) in the red oak smoke samples. [file 12989_2024_575_MOESM7_ESM.docx]

**Supplemental Table 1.**

Concentrations (ppbv) of volatile organic compounds (N=154) in the red oak smoke samples.

|  | MDL Conc (ppbv) | Sample 1 Conc (ppbv) | Sample 2 Conc (ppbv) |
| --- | --- | --- | --- |
| Methanol | 5.14 | 39198.45 | 33992.97 |
| Acetaldehyde | NR | 5831.58 | 3146.92 |
| Formaldehyde | NR | 4947.85 | 2544.97 |
| 3-Furaldehyde | 0.05 | 4097.05 | 1746.53 |
| 2-Methylfuran | 0.05 | 2076.35 | 1584.78 |
| Acrolein | 0.04 | 1994.64 | 1197.06 |
| Propylene | 0.03 | 1830.71 | 1763.17 |
| Acetone | 0.13 | 1796.22 | 1471.87 |
| Furan | 0.02 | 967.87 | 683.44 |
| 2-Butanone | 0.04 | 806.02 | 139.27 |
| Propanal | 0.23 | 799.29 | 502.87 |
| Methyl Vinyl Ketone | 0.03 | 704.76 | 121.66 |
| Propane | 0.07 | 680.2 | 577.41 |
| Crotonaldehyde | 0.05 | 668.07 | 0 |
| 3-Methyl-3-Butene-2-one | 0.03 | 614.25 | 88.64 |
| 1-Butene | 0.01 | 451.75 | 549.28 |
| 2,5-Dimethylfuran | 0.03 | 400.97 | 267.11 |
| Benzene | 0.01 | 345.06 | 249.02 |
| Methacrolein | 0.02 | 261.37 | 212.53 |
| Toluene | 0.02 | 230.97 | 158.04 |
| 1,3-Butadiene | 0.01 | 228.02 | 181.67 |
| 2-Furaldeyde | 0.03 | 172.75 | 44.24 |
| 3-Methylfuran | 0.03 | 141.58 | 88.42 |
| Acetonitrile | 0.13 | 125.33 | 159.99 |
| Butanal | 0.17 | 104.97 | 0 |
| Ethanol | 0.26 | 103.26 | 0 |
| Isoprene | 0.01 | 85.24 | 56.37 |
| Heptane | 0.02 | 82.47 | 19.24 |
| Ethylene oxide 44 | 0.02 | 79.26 | 33.58 |
| Butane | 0.05 | 78.61 | 77.29 |
| 2-Ethylfuran | 0.03 | 70.3 | 49.74 |
| mp-Xylene | 0.02 | 68 | 37.41 |
| Ethylene oxide 29 | 0.02 | 67.79 | 58.53 |
| Ethyl acrylate | 0.02 | 61.68 | 0 |
| cis-2-butene | 0.01 | 55.78 | 53.28 |
| trans-2-butene | 0.01 | 54.38 | 53.41 |
| Benzaldehyde | NR | 51.77 | 25.26 |
| p-tolualdehyde | 0.05 | 48.14 | 0 |
| Benzofuran | 0.04 | 35.86 | 0 |
| Styrene | 0.03 | 34.38 | 0 |
| Tetrahydrofuran | 0.02 | 33.99 | 0 |
| trans-2-pentene | 0.01 | 30.77 | 31.14 |
| o-Xylene | 0.02 | 28.04 | 0 |
| Ethylbenzene | 0.02 | 27.43 | 0 |
| 1-Pentene | 0.01 | 25.96 | 22.76 |
| 1-Hexene | 0.01 | 24.18 | 0 |
| cis-2-pentene | 0.01 | 23 | 14.37 |
| Isobutane | 0.01 | 22.56 | 24.46 |
| mo-Tolualdehyde | 0.11 | 21.26 | 0 |
| Hexanal | 0.05 | 20.65 | 0 |
| Isopropyl Alcohol | 0.04 | 18.88 | 0 |
| n-Pentane | 0.01 | 17.95 | 8.75 |
| 2-Hexanone | 0.02 | 16.92 | 0 |
| 2-Methylbenzofuran | 0.05 | 16.75 | 0 |
| Pentanal | 0.04 | 13.92 | 0 |
| o-Ethyltoluene | 0.03 | 10.79 | 0 |
| Chloromethane | 0.01 | 10.66 | 7.58 |
| Vinyl Acetate | 0.01 | 10.65 | 0.32 |
| n-Hexane | 0.01 | 10.28 | 0 |
| Methyl Methacrylate | 0.02 | 9.99 | 0 |
| Naphthalene | 0.04 | 8.53 | 0 |
| Heptanal | 0.09 | 8.51 | 0 |
| m-Ethyltoluene | 0.02 | 8.38 | 0 |
| 1,2-Dichloroethane | 0 | 6.88 | 4.08 |
| iso-Pentane | 0.02 | 6.37 | 3.19 |
| Acrylonitrile | 0.02 | 6.22 | 0 |
| Octane | 0.02 | 5.01 | 0 |
| Methylcyclopentane | 0.01 | 3.55 | 0 |
| 2-Methylpentane | 0.01 | 3.26 | 0 |
| Nonane | 0.03 | 3.22 | 0 |
| 2,3,4-Trimethylpentane | 0.04 | 2.85 | 0 |
| 2,3-Dimethylbutane | 0.01 | 2.7 | 0 |
| 4-Methy-2-Pentanone | 0.02 | 2.48 | 0 |
| 1,2-Dichloropropane | 0.01 | 2.12 | 0 |
| Cyclopentane | 0.01 | 1.97 | 0 |
| 3-Methylpentane | 0.01 | 1.89 | 0 |
| 2,3-Dimethylpentane | 0.01 | 1.88 | 0 |
| 2-Methylheptane | 0.02 | 1.74 | 0 |
| Ethyl Acetate | 0.01 | 1.68 | 0 |
| 3-Methylheptane | 0.02 | 1.52 | 0 |
| 3-Methylbenzofuran | 0.04 | 1.45 | 0 |
| n-Propylbenzene | 0.02 | 0.79 | 0 |
| Chloroethane | 0 | 0.58 | 0 |
| 1,4-Dioxane | 0.02 | 0.49 | 0 |
| Tert-Butanol | 0.02 | 0.43 | 0 |
| 2-Methylhexane | 0.01 | 0.42 | 0 |
| Trichlorofluoromethane | 0 | 0.03 | 0 |
| Octanal | 0.19 | 0 | 0 |
| Nonanal | 0.47 | 0 | 0 |
| 2,5-Dimethylbenzaldehyde | 0 | 0 | 0 |
| Dichlorodifluoromethane | 0.01 | 0 | 0 |
| Dichlorotetrafluoroethane | 0 | 0 | 0 |
| Vinyl Chloride | 0.01 | 0 | 0 |
| Bromomethane | 0 | 0 | 0 |
| Vinyl Bromide | 0 | 0 | 0 |
| 1,1-Dichloroethene | 0.01 | 0 | 0 |
| 1,1,2-Trichloro-1,2,2-... | 0 | 0 | 0 |
| 2,2-Dimethylbutane | 0.09 | 0 | 0 |
| Carbon Disulfide | 0.22 | 0 | 0 |
| 3-Chloro-1-Propene | 0.01 | 0 | 0 |
| Methylene Chloride | 0 | 0 | 0 |
| trans-1,2-Dichloroethene | 0.01 | 0 | 0 |
| Methyl Tert-Butyl Ether | 0.01 | 0 | 0 |
| 1,1-Dichloroethane | 0 | 0 | 0 |
| 2-Chloroprene | 0.01 | 0 | 0 |
| Diisopropyl Ether | 0.01 | 0 | 0 |
| 2,4-Dimethylpentane | 0.04 | 0 | 0 |
| Ethyl Tert-Butyl Ether | 0.01 | 0 | 0 |
| cis-1,2-Dichloroethene | 0.02 | 0 | 0 |
| Chloroform | 0.01 | 0 | 0 |
| 1,1,1-Trichloroethane | 0 | 0 | 0 |
| Cyclohexane | 0.01 | 0 | 0 |
| Carbon Tetrachloride | 0 | 0 | 0 |
| 3-Methylhexane | 0.03 | 0 | 0 |
| Isooctane | 0.01 | 0 | 0 |
| Tert Amyl-Methyl Ether | 0.01 | 0 | 0 |
| Trichloroethene | 0.01 | 0 | 0 |
| Methylcyclohexane | 0.01 | 0 | 0 |
| Bromodichloromethane | 0.01 | 0 | 0 |
| cis-1,3-Dichloropropene | 0.01 | 0 | 0 |
| trans-1,3-Dichloropropene | 0.02 | 0 | 0 |
| 1,1,2-Trichloroethane | 0 | 0 | 0 |
| Tetrachloroethene | 0 | 0 | 0 |
| Dibromochloromethane | 0.01 | 0 | 0 |
| 1,2-Dibromoethane | 0.01 | 0 | 0 |
| Chlorobenzene | 0.01 | 0 | 0 |
| 1,1,1,2-Tetrachloroethane | 0.01 | 0 | 0 |
| Butyl Acrylate | 0.03 | 0 | 0 |
| Bromoform | 0.02 | 0 | 0 |
| a-Pinene | 0.03 | 0 | 0 |
| Cumene | 0.02 | 0 | 0 |
| Bromofluorobenzene | 0 | 0 | 0 |
| 1,1,2,2-Tetrachloroethane | 0.01 | 0 | 0 |
| Chlorotoluenes | 0.02 | 0 | 0 |
| 1-Ethyl-4-Methyl Benzene | 0.03 | 0 | 0 |
| 1,3,5-Trimethylbenzene | 0.02 | 0 | 0 |
| n-Decane | 0.03 | 0 | 0 |
| b-Pinene | 0.03 | 0 | 0 |
| 1,2,4-Trimethylbenzene | 0.03 | 0 | 0 |
| Tert-Butyl Benzene | 0.03 | 0 | 0 |
| Sec-Butyl Benzene | 0.03 | 0 | 0 |
| 1,3-Dichlorobenzene | 0.02 | 0 | 0 |
| 1,4-Dichlorobenzene | 0.02 | 0 | 0 |
| 1,2,3-Trimethylbenzene | 0.03 | 0 | 0 |
| o-Cymene | 0.03 | 0 | 0 |
| 1,3-Diethylbenzene | 0.03 | 0 | 0 |
| 1,2-Diethylbenzene | 0.03 | 0 | 0 |
| n-Butyl Benzene | 0.03 | 0 | 0 |
| 1,2-Dichlorobenzene | 0.02 | 0 | 0 |
| Undecane | 0.04 | 0 | 0 |
| Dodecane | 0.07 | 0 | 0 |
| 1,2,4-Trichlorobenzene | 0.03 | 0 | 0 |
| Decanal | 0.03 | 0 | 0 |
| Hexachlorobutadiene | 0.02 | 0 | 0 |
