# Supplementary material for: Monitoring redox stress in human airway epithelial cells exposed to woodsmoke at an air–liquid interface
Source: Part Fibre Toxicol. 2024 Mar 8;21:14. doi: 10.1186/s12989-024-00575-9 (PMC10921608; doi:10.1186/s12989-024-00575-9)
Supplement: Supplementary file 8 — Additional file 8. Table S2. Semi-volatile organic compounds (N = 88) measured in two red oak smoke samples collected on separate days. * Compound could not be unambiguously identified (N.D.); ** Values below reliable quantification (> Quant) [file 12989_2024_575_MOESM8_ESM.docx]

**Supplemental Table 2.**

Semi-volatile organic compounds (N=88) measured in two red oak smoke samples collected on separate days.

|  | Concentration (ng) | |
| --- | --- | --- |
|  | Sample 1 | Sample 2 |
| Isoeugenol | 728.57 | 457.82 |
| Syringealdehyde | 467.01 | 811.45 |
| Vanillin | 213.49 | 152.36 |
| 3,5-Dimethoxy-4-hydroxyphenol | 128.74 | 247.45 |
| 4-Hydroxy-3-Methoxy Cinnamaldehyde | 37.09 | 91.43 |
| Guaiacol | 22.4 | 22.96 |
| Ethylguaiacol | 18.94 | 11.3 |
| 2-Methoxymethylphenol | 18.84 | 11.84 |
| Eugenol | 11.12 | 6.94 |
| Propylguaiacol | 9.49 | 5.93 |
| Phenol | 7.24 | 4.96 |
| 17A(H)-22,29,30-Trisnorhopane | 3.75 | N.D. |
| Docosane | 3.73 | 2.11 |
| Tricosane | 3.66 | 2.13 |
| Tritriacontane | 3.29 | 1.23 |
| Tetratriacontane | 3.07 | N.D.* |
| m,p-Cresols | 3.02 | 0.89 |
| Pentatriacontane | 2.84 | 1.6 |
| Triacontane | 2.76 | 1.93 |
| Nonacosane | 2.68 | 1.78 |
| Dotriacontane | 2.58 | N.D. |
| o-Cresol | 2.49 | 0.84 |
| Hentriacontane | 2.38 | 0.98 |
| Octacosane | 2.21 | 1.88 |
| Heneicosane | 2.14 | 1.41 |
| Heptacosane | 2.08 | 1.96 |
| Pentacosane | 2.04 | 1.79 |
| Eicosane | 1.58 | 1 |
| Tetracosane | 1.56 | 1.13 |
| Hexacosane | 1.52 | 1.47 |
| Squalane | 1.39 | N.D. |
| Pyrene | 0.42 | 0.36 |
| Chrysene | N.D. | 0.3 |
| Benz(a)anthracene | N.D. | 0.19 |
| Naphthalene | N.D. | N.D. |
| 1-Methylnapthalene | N.D. | N.D. |
| 2-Methylnapthalene | N.D. | N.D. |
| 2,6-Dimethylnapthalene | N.D. | N.D. |
| Acenaphthylene | N.D. | N.D. |
| Acenaphthene | N.D. | N.D. |
| Dibenzofuran | N.D. | N.D. |
| Fluorene | N.D. | N.D. |
| Methylfluorene | N.D. | N.D. |
| Phenanthrene | N.D. | N.D. |
| Anthracene | N.D. | N.D. |
| 9-Methylanthracene | N.D. | N.D. |
| Fluoranthene | N.D. | N.D. |
| Retene | N.D. | N.D. |
| Decane | N.D. | N.D. |
| Undecane | N.D. | N.D. |
| Dodecane | N.D. | N.D. |
| Tridecane | N.D. | N.D. |
| Tetradecane | N.D. | N.D. |
| Pentadecane | N.D. | N.D. |
| Hexadecane | N.D. | N.D. |
| Heptadecane | N.D. | N.D. |
| Octadecane | N.D. | N.D. |
| Phytane | N.D. | N.D. |
| Dodecylcyclohexane | N.D. | N.D. |
| Nonadecane | N.D. | N.D. |
| 2-Methylnonadecane | N.D. | N.D. |
| 3-Methylnonadecane | N.D. | N.D. |
| Pentadecylcyclohexane | N.D. | N.D. |
| Nonadecylcyclohexane | N.D. | N.D. |
| Pristane | N.D. | N.D. |
| Benzo(ghi)fluoranthene | N.D. | N.D. |
| Cyclopenta(cd)pyrene | N.D. | N.D. |
| 1-Methylchysene | N.D. | N.D. |
| Benzo(b)fluoranthene | N.D. | N.D. |
| Benzo(k)fluoranthene | N.D. | N.D. |
| Benzo(e)pyrene | N.D. | N.D. |
| Benzo(a)pyrene | N.D. | N.D. |
| Perylene | N.D. | N.D. |
| Indeno[1,2,3-cd] pyrene | N.D. | N.D. |
| Dibenzo[a,h]anthracene | N.D. | N.D. |
| Benzo[ghi]perylene | N.D. | N.D. |
| Coronene | N.D. | N.D. |
| Dibenzo(a,e)pyrene | N.D. | N.D. |
| Cholestane | N.D. | N.D. |
| AAA-20S-Cholestame | N.D. | N.D. |
| ABB-Methylcholestane | N.D. | N.D. |
| ABBEthylcholestane | N.D. | N.D. |
| 17B(H)-21A(H)-30-Norhopane | N.D. | N.D. |
| 17A-(H-21B(H)-Hopane | N.D. | N.D. |
| 3,5-Dimethoxyphenol | N.D. | N.D. |
| Hexatriacontane | <Quant | <Quant |
| Heptatriacontane | <Quant | <Quant |
| Octatricontane | <Quant | <Quant |

* Compound could not be unambiguously identified (N.D.)

** Values below reliable quantification (>Quant)
