# Supplementary material for: Monitoring redox stress in human airway epithelial cells exposed to woodsmoke at an air–liquid interface
Source: Part Fibre Toxicol. 2024 Mar 8;21:14. doi: 10.1186/s12989-024-00575-9 (PMC10921608; doi:10.1186/s12989-024-00575-9)
Supplement: Supplementary file 9 — Additional file 9. Table S3. Concentration (ppm) of inorganic elemental constituents of red oak smoke. *Elemental concentrations of Zn, Ni, Mn, V, Cu, and Mg are not detectable [file 12989_2024_575_MOESM9_ESM.docx]

**Supplemental Table 3.**

Concentration (ppm) of inorganic elemental constituents of red oak smoke.

| Metal | Concentration (ppm) |
| --- | --- |
| Fe | 1.83 ± 0.03 |
| Ca | 0.99 ± 0.02 |
| K | 0.15 ± 0.02 |
| Cr | 0.62 ± 0.01 |

*Elemental concentrations of Zn, Ni, Mn, V, Cu, and Mg are not detectable
